# Low-Grade Fibromyxoid Sarcoma of the Back

**DOI:** 10.7759/cureus.17308

**Published:** 2021-08-19

**Authors:** Nicholas A Cantu, Asad Ullah, Lorie Stumpo-Decoons, Sami Belakhlef, Edward J Kruse

**Affiliations:** 1 Pathology, Medical College of Georgia - Augusta University, Augusta, USA; 2 Radiology, Medical College of Georgia - Augusta University, Augusta, USA; 3 Surgery, Medical College of Georgia - Augusta University, Augusta, USA

**Keywords:** lipoma, low-grade fibromyxoid sarcoma, magnetic resonance imaging, soft tissue tumours, metastasis

## Abstract

A 29-year-old male presented with a seven-year history of a slow-growing, painless, firm, mobile mass in the right upper back that was bothersome when supine or with direct pressure. On initial presentation, a clinical diagnosis of lipoma was given. The mass progressively increased in size over several years but remained painless. The mass measured 15 x 10 cm on examination. Excision of the lesion was performed, which revealed a white cut surface with cystic degenerative changes. Histologically, the lesion revealed spindle cell morphology with occasional mitosis. Diffuse immunohistochemical staining with MUC4 supports a diagnosis of low-grade fibromyxoid sarcoma (LGFMS). Tumor was present with focal extension into the deep margin. However, serial magnetic resonance imaging studies performed suggest no residual disease and negative regional lymph node involvement. This case demonstrates the growth pattern of LGFMS, but also denotes the importance of correlating radiological and pathological features to accurately diagnose and treat these tumors in a timely fashion.

## Introduction

Low-grade fibromyxoid sarcoma (LGFMS) is a rare form of malignant tumor that often presents as a painless, slow-growing mass in the deep soft tissues of the trunk or proximal extremities of young adults [1,2]. This tumor was first described by Dr. Harry Evans in 1987 [2]. Previous studies have described LGFMS to represent about 0.6% of all soft-tissue sarcomas, with an incidence of 0.18 per million. The LGFMS commonly presents as bland-appearing, slender spindle cells in a whorling pattern that contains a mixture of collagenized and myxoid areas with inconspicuous nucleoli and scant, wispy cytoplasm [3]. The characteristic pattern of immunostaining for LGFMS shows strong, diffuse granular cytoplasmic immunoreactivity with MUC4. Due to the benign and heterogeneous appearance on histology, the diagnosis of LGFMS can be challenging [1]. LGFMS has shown translocation between chromosomes 7 and 16, resulting in a chimeric fusion protein derived from the fused in sarcoma (FUS) gene of chromosome 16p11 and the cAMP-responsive element-binding protein 3-like 2 (CREB3L2) gene of 17q33. A minority of cases have been shown to display a FUS-CREB3L1 [3]. Despite this low-grade appearance, LGFMS has shown potential for late metastasis. This makes these lesions difficult to diagnose on small biopsy specimen and to excise with clean margins. There is no clear guidance currently in the literature on how to best manage LGFMS, likely due to the rare nature of these tumors. While the traditional first-line treatment of soft-tissue tumors is wide surgical resection, there seems to be a lack of data on the efficacy of adjuvant chemotherapy and/or radiotherapy [1].

## Case presentation

A 29-year-old male with no significant past medical history presented to his primary care provider for an enlarging mass on his upper back, inferior and lateral to the right scapula. He complained of discomfort when lying in the supine position and with direct pressure on the mass. He otherwise had no local pain, erythema, warmth, or drainage from the mass. This was originally noticed by the patient about seven years prior and was described as firm, mobile and about 5 cm in diameter. He was given a working diagnosis of lipoma pending further workup. After referral to dermatology, the mass was noted to be firmer than a characteristic lipoma, and the patient was scheduled for excision with the department of surgery. At that time, the patient was lost to follow up. On return visit seven years later, he presented with the complaint that the mass had grown. On examination, the mass remained firm, mobile, and non-fluctuant.

**Figure 1:**
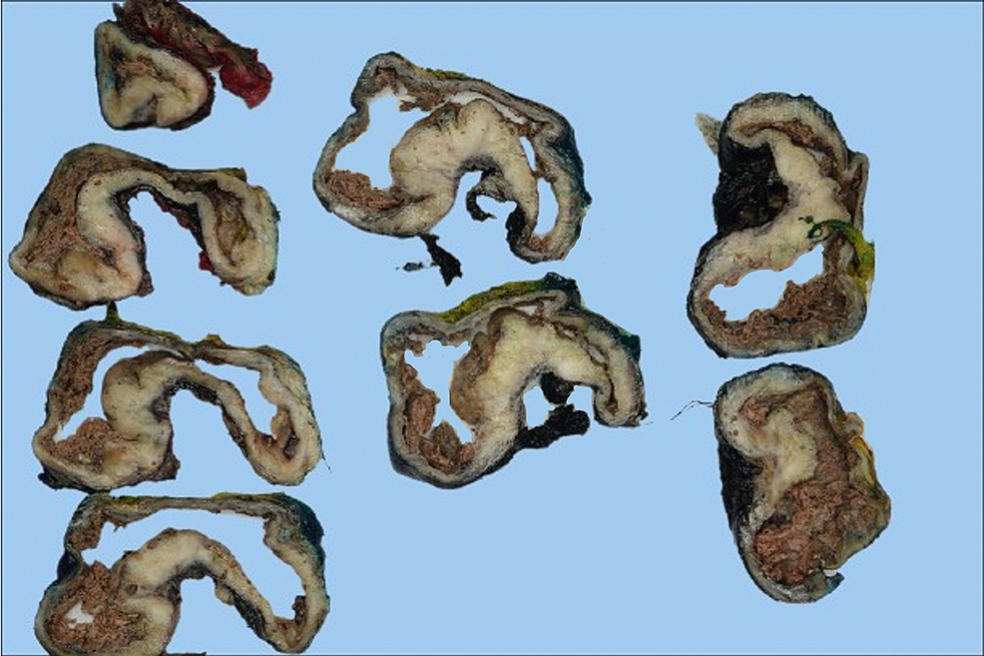
Circumscribed lesion with a central cystic area filled with necrotic material and a peripheral rim of tan-white solid lesion extending to the ink margin

**Figure 2:**
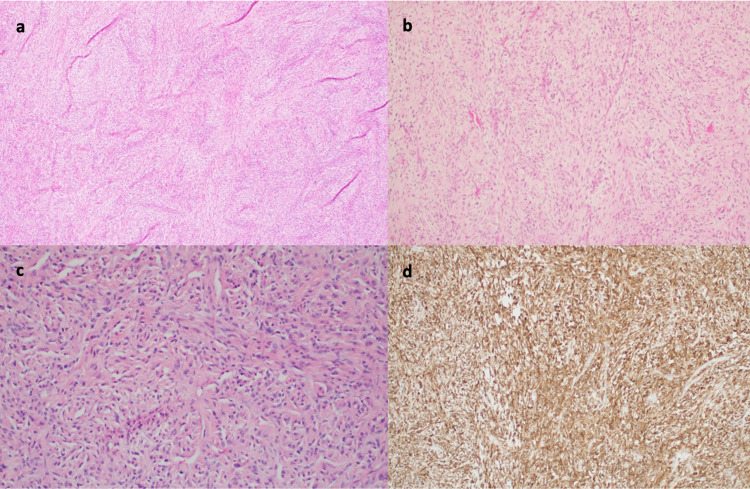
Pathologic specimen of back mass (a,b,c) H&E stain showing (a, 4X) spindle cell proliferation with fibrous and myxoid areas, (b, 10X) hypocellular areas with curvilinear vessels, and (c, 20X) monomorphic cells with bland cytology. (d) MUC4, diffuse and strong staining of lesion.

Surgical excision of the mass resulted in retrieval of a 15 x 10 cm cystic mass adherent to the superior portion of the trapezius muscle, but non-adherent to deeper structures or the scapula. On gross examination, the outer surface was tan-white and smooth. Serial sectioning revealed a tan-white cut surface with cystic degeneration (Figure 1). No hemorrhage or necrosis was identified. Histologic sectioning revealed a spindle cell proliferation with alternating hyper- and hypocellular areas in a fibrous and myxoid background. The spindle cells are relatively monotonous with small hyperchromatic oval nuclei and occasional small nucleoli and eosinophilic cytoplasm. Prominent curvilinear vessels and rare mitoses were present. No cytologic atypia or necrosis was identified. Immunohistochemistry demonstrated that the neoplastic cells are strong and diffusely positive for MUC4 (Figure 2) and negative for S100, SOX-10, pankeratin, smooth muscle actin, desmin, and CD34. Based on histology and immunohistochemical profile, diagnosis of LGFMS was rendered.

**Figure 3:**
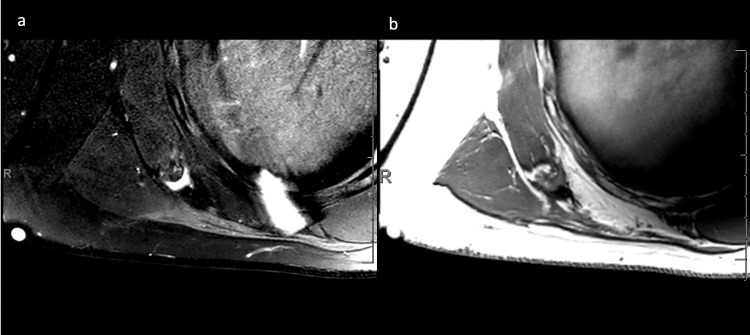
Post-resection axial magnetic resonance imaging of back mass Axial post-contrast T2 SPAIR (a) and axial T1 (b) MR images of right upper back status post resection of soft-tissue mass. Focal fluid collection measured to be approximately 1.4 x 0.5 x 2.3 cm, which was noted to be decreased in size from previous imaging study. SPAIR, spectral attenuated inversion recovery; MR, magnetic resonance.

Upon pathological analysis, the deep margin was positive for the tumor. This case was discussed by a multidisciplinary cancer tumor board and close follow-up with imaging was recommended. Post-operative magnetic resonance imaging (MRI) at one month showed focal nodular enhancement within a small fluid collection and nodularity thought to be either post-surgical change or residual tumor (Figure 3). The patient then returned for short-interval imaging at four months after resection, which showed interval decrease in fluid collection and resolution of nodularity, favoring post-surgical changes with no evidence of local recurrence. It was recommended that the patient follow up with imaging every six months going forward.

## Discussion

LGFMS is a potentially challenging diagnosis, as its indolent growth pattern and benign appearance on small biopsy could be mistaken for benign soft-tissue tumors due to its late metastatic potential [1,2]. Most commonly, LGFMS is seen in young adults and is located in the extremities or trunk [3]. Imaging studies are unable to provide definitive diagnosis, and, therefore, biopsy and histopathologic diagnosis are required. According to Maretty-Nielsen et al., current treatment of low-grade sarcomas is surgical excision without adjuvant therapy, as low-grade tumors often do not respond to chemotherapy and their recurrence, if any, is typically local [1].

Regarding potential for late metastasis in LGFMS, this is most commonly seen with involvement of the lungs. Of note, in Evans' (2011) study following 33 LGFMS cases with long-term follow-up, metastasis occurred in half of the patients and there was a 42% mortality rate [4].

The main differential diagnosis of LGFMS includes myxofibrosarcoma (MFS), dermatofibrosarcoma protuberans (DFSP), desmoid fibromatosis, nodular fasciitis, malignant peripheral nerve sheath tumor (MPNST), and sclerosing epithelioid fibrosarcoma (SEF).

MFS is a malignant fibroblastic tumor typically found in elderly populations. It is commonly located in the dermis and subcutis of extremities. MFS usually has abundant myxoid stroma and less fibrous areas compared to LGFMS. MFS is also pleomorphic and hyperchromatic compared to LGFMS, which has comparatively bland cytology. Low-grade MFS most closely mimics the presentation of LGFMS. Due to high risk of local recurrence, treatment generally includes adjuvant radiotherapy to improve outcomes [5,6].

DFSP typically presents as an asymptomatic, slow-growing violaceous nodule or plaque. Highest incidence is noted in patients 30-50 years old, and incidence is nearly double among blacks compared to white patients. These tumors are often asymmetric, and detailed mapping has shown long tentacle-like projections that extend beyond 3 cm [6]. For this reason, curative treatment of localized DFSP is typically done with Mohs micrographic surgery (MMS) or wide local excision if MMS is not available [7].

LGFMS may also show areas histologically similar to desmoid fibromatosis. These tumors, however, are not well circumscribed, show infiltrative borders, and exhibit myofibroblastic differentiation. There is a significant association of these tumors with familial adenomatous polyposis. Surgical intervention with active surveillance has now been suggested as a reasonable initial strategy in most patients with desmoid fibromatosis. Other non-operative options include nonsteroidal anti-inflammatory drugs, hormone therapy, cytotoxic chemotherapy, and targeted agents [8].

Nodular fasciitis is a benign tumor that often originates in the extremities, head, neck, and trunk. These tumors are most often also solitary, well-circumscribed lesions found in adults 20-40 years old. Diagnosis can be aided by positive staining for alpha-smooth muscle actin. Simple excision is the most common treatment and post-resection recurrence is noted to be very rare [9].

MPNST is yet another form of soft-tissue sarcoma. One-half of these tumors arise in patients with neurofibromatosis type I, and these tumors may be preceded by peripheral neuromas or atypical neurofibromas. They often show positive staining for S100, which is not present in LGFMS. While workup and treatment of these precursor tumors vary, once diagnosed with MPNST, complete surgical resection with wide tissue margins is required. Chemotherapy and radiation therapy may also be done as indicated [5].

SEF is a diagnosis that has been thought to exist on a spectrum with LGFMS, but represents a much more aggressive tumor with high rates of recurrence and metastasis. Patients with SEF are often of older age, found in the lower extremity, and have involvement of bone. Due to the rarity of the tumor, no clear treatment guidelines have been established, but amputation, wide excision, radio- and chemotherapy, and various combinations have been attempted [10].

## Conclusions

LGFMS is a slow-growing soft-tissue tumor with low-grade morphology, but metastatic potential. Thus, clinicopathologic correlation and adequate sampling are required for proper diagnosis. Long-term radiologic follow-up is also needed to monitor for metastasis.
